# Data-Driven Quantitative Structure–Activity Relationship Modeling for Human Carcinogenicity by Chronic Oral Exposure

**DOI:** 10.1021/acs.est.3c00648

**Published:** 2023-04-11

**Authors:** Elena Chung, Daniel P. Russo, Heather L. Ciallella, Yu-Tang Wang, Min Wu, Lauren M. Aleksunes, Hao Zhu

**Affiliations:** † Department of Chemistry and Biochemistry, Rowan University, 201 Mullica Hill Road, Glassboro, New Jersey 08028, United States; ‡ Department of Toxicology, Cuyahoga County Medical Examiner’s Office, 11001 Cedar Avenue, Cleveland, Ohio 44106, United States; § Institute of Agro-Products Processing Science and Technology, Chinese Academy of Agricultural Sciences/Key Laboratory of Agro-Products Processing, Ministry of Agriculture, Beijing 100193, China; ∥ School of Life Science and Technology, China Pharmaceutical University, No. 24, Tong Jia Xiang, Nanjing 210009, China; ⊥ Department of Pharmacology and Toxicology, Rutgers University, Ernest Mario School of Pharmacy, 170 Frelinghuysen Road, Piscataway, New Jersey 08854, United States

**Keywords:** quantitative structure−activity relationships, models, carcinogens, big data, data mining, machine learning

## Abstract

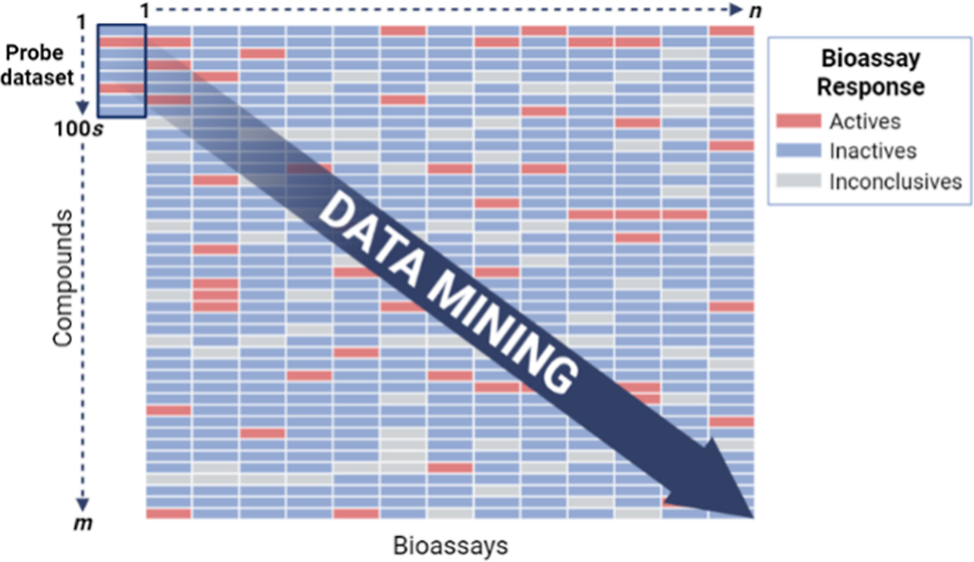

Traditional methodologies for assessing chemical toxicity are expensive and time-consuming. Computational modeling approaches have emerged as low-cost alternatives, especially those used to develop quantitative structure–activity relationship (QSAR) models. However, conventional QSAR models have limited training data, leading to low predictivity for new compounds. We developed a data-driven modeling approach for constructing carcinogenicity-related models and used these models to identify potential new human carcinogens. To this goal, we used a probe carcinogen dataset from the US Environmental Protection Agency’s Integrated Risk Information System (IRIS) to identify relevant PubChem bioassays. Responses of 25 PubChem assays were significantly relevant to carcinogenicity. Eight assays inferred carcinogenicity predictivity and were selected for QSAR model training. Using 5 machine learning algorithms and 3 types of chemical fingerprints, 15 QSAR models were developed for each PubChem assay dataset. These models showed acceptable predictivity during 5-fold cross-validation (average CCR = 0.71). Using our QSAR models, we can correctly predict and rank 342 IRIS compounds’ carcinogenic potentials (PPV = 0.72). The models predicted potential new carcinogens, which were validated by a literature search. This study portends an automated technique that can be applied to prioritize potential toxicants using validated QSAR models based on extensive training sets from public data resources.

## Abstract 2

A computational approach that expands a small dataset can be used to develop predictive models for complex toxicity endpoints, such as human carcinogenicity, supporting the urgent requests for regulatory actions that minimize animal testing in safety evaluations.

## Introduction

Humans are exposed to various toxicants daily, leading to adverse health effects. Morbidity and mortality from environmental contaminants critically impact human health.^1−5^ Among the current consumer products on the market, there are over 100,000 compounds that lack sufficient information to evaluate their toxicity potential to humans.^6,7^ Most traditional toxicity testing was performed *in vivo*, including preclinical and clinical evaluations of potential carcinogens. These costly traditional toxicity models are low-throughput and labor-intensive. Thus, alternative approaches to prioritize potentially toxic compounds for further experimental evaluations could significantly advance the current chemical risk assessment procedure by reducing the time and economic burden of evaluating new compounds for potential health risks.^8,9^

A carcinogen is a chemical that can cause cancer. Unfortunately, the carcinogenic potential of most available compounds remains unknown due to limited available data. In 1985, the United States Environmental Protection Agency (EPA) launched its publicly available and annually updated Integrated Risk Information System (IRIS) database (https://www.epa.gov/iris/), which characterizes 485 compounds by toxicity assessments related to human carcinogenicity to date.

Computational modeling, such as that based on quantitative structure–activity relationships (QSARs), is a powerful tool to evaluate new compounds’ toxicities from their chemical structures directly.^10^ Cost-effective QSAR models can prioritize compounds for experimental testing and improve efficiency by virtually screening large databases.^11−14^ QSAR approaches were also used to develop models for chemical carcinogens.^15−18^ For example, Toma et al. recently developed QSAR models from oral and inhalation slope factors, representing carcinogenicity potency from a toxicity database.^18^ However, due to the limited known human carcinogens, previous QSAR studies were established on a limited number of compounds (i.e., small training sets). Because the applicability of resulting models relies on the chemical space covered by the training set compounds, model developments with small training sets cannot predict most new compounds well. On the other hand, the prohibitive cost of animal testing and clinical studies limits the availability of new data on complex chemical toxicity endpoints (e.g., carcinogenicity). To directly address this challenge, data-driven approaches can gather large training sets from various sources to significantly increase model coverages, often involving an automatic mining and curation process.^19−24^

In this study, we developed a data-driven QSAR modeling approach that can be applied to expand training sets for target toxicity modeling significantly. As a result, the models can be developed with more training data. To this end, chemical carcinogenicity in humans was selected for modeling. The initial probe training set was obtained from the US EPA’s IRIS database, including human carcinogenicity classifications. An in-house tool automatically searched for all available toxicity data statistically relevant to human carcinogenicity using the probe dataset.^25^ Hundreds of QSAR models were developed using an automatic modeling workflow using the optimized assay data pertinent to human carcinogenicity. Compared to other existing data-driven models of chemical toxicities, which require biological data of new compounds for predictive purposes,^26−28^ this study presents a modeling strategy that is more suitable for the virtual screening of new compounds based on chemical structure information. The prioritization of potential carcinogens by screening new compounds demonstrates the applicability of this data-driven modeling approach. Our results suggest that this approach can effectively organize a more extensive training set to maximize overall chemical diversity, facilitate model developments, and predict new compounds.

## Methods

### Training Set

The probe training set was obtained from the EPA’s IRIS database (https://www.epa.gov/iris/, accessed January 10, 2022), consisting of 485 compounds.^29^ The IRIS database is appropriate for this proof-of-concept research that contains a valuable collection of well-studied human carcinogens. Therefore, the data mining procedure described below can ensure the extraction of sufficient bioassay data for modeling purposes. The initial IRIS database compounds consisted of two assessment classifications: human carcinogens or noncarcinogens and two toxicity value types: oral slope factor (OSF) and reference dose. These factors were scrutinized for each compound. For this study, human carcinogens were only derived from the OSF, a key risk assessment parameter to estimate cancer risk by oral intake.^30^ The probe training set was curated using the CASE Ultra v1.8.0.4 DataKurator tool (MultiCASE Inc., Beachwood, OH) to delete duplicates and inorganic compounds and reserve the largest organic counterpart of the neutralized salt compounds. The remaining 342 unique compounds were used as the probe for data mining and constructing models (Supporting Information Table S1).

The assays containing the test results for the 342 probe training set compounds were automatically mined from PubChem (https://pubchem.ncbi.nlm.nih.gov/) using an in-house data profiling tool.^25^ Briefly, all 342 compounds were used as a probe set to search for their bioassay responses from PubChem, and their response profiles were represented by their PubChem bioassay testing outcome classifications (i.e., active, inactive, or inconclusive). PubChem assays relevant to carcinogenicity were selected using (1) the number of probe compounds tested in a PubChem assay, (2) the number of active responses across these compounds, and (3) the statistical significance between chemical carcinogenicity and PubChem assay responses.

### External Datasets

Five datasets were compiled to validate the predictivity of the resulting QSAR models. These datasets are collections of different types of compounds. A pesticide database was established by retrieving information from the literature^14,31^ and public databases.^32−35^ This pesticide database originally included 1741 compounds and contained 1009 unique compounds after conducting the structure curation described above. The cosmetics dataset, retrieved from the COSMOS Cosmetics Inventory, initially comprised 5280 compounds and 4129 unique compounds after data curation.^36^ The high-production volume chemical database (https://comptox.epa.gov/dashboard/chemical-lists/EPAHPV/, accessed January 10, 2022) of 2891 compounds underwent curation and comprised 1672 unique compounds. The natural product database contained 6527 compounds, and 2479 unique compounds remained after the curation collected from the traditional Chinese medicine systems pharmacology database and analysis platform.^37^ The drug database retrieved from DrugBank (https://www.drugbank.com/, accessed January 10, 2022) consisted of 8696 and 8055 unique compounds before and after data curation, respectively.^38^

### QSAR Model Development

Five machine learning (ML) algorithms were used for QSAR model development, including the AdaBoost decision tree (ADA), Bernoulli Naïve Bayes (BNB), *k*-nearest neighbors (*k*NNs), random forest (RF), and support vector machines (SVMs). All five ML algorithms were implemented in Python v3.9.4 using scikit-learn v0.24.1 (http://scikit-learn.org/) within a publicly available QSAR modeling workflow.^39,40^ With an adaptive approach, ADA models combine decision trees to correct poorly predicted training data points by normalizing their weights.^41,42^ BNB models, based on Bayes’ theorem, assume that all descriptors are independent, where one descriptor does not offer information about another, maximizing the joint likelihood.^43^*k*NN models rely on the *k*-nearest neighbors, defined by subspace chemical similarities, to predict the chemical activity of the target compound.^44^ RF models use an ensemble learning method to construct decision trees after randomly selecting targets and features in the training set.^45^ SVM models optimize thresholds for each descriptor that best separates active and inactive training compounds.^46^ During model development, tunable parameters for each ML algorithm were optimized to fit the training data, as described in our previous studies.^21,47,48^

Three types of chemical fingerprints were implemented within the cheminformatics package RDKit v2021.03.1 (http://www.rdkit.org/) using Python v3.9.4, including Molecular ACCess System (MACCS), extended-connectivity fingerprints (ECFPs), and functional-class fingerprints (FCFPs), generated for all compounds.^49^ The MACCS keys are 166 bit two-dimensional substructure fingerprints. The ECFPs and FCFPs are both 1024 bit length binary vectors, which obtain the atom properties and characterize the general functional roles of the atom, respectively. ECFPs and FCFPs were generated using a bond radius of 3, as successfully applied in earlier computational toxicology studies.^47^

Cross-validation procedures that partition compounds on different iterations infer reliable model evaluations.^50^ In this study, all models were evaluated using a 5-fold cross-validation procedure.^51^ Briefly, a training set was randomly split into five equivalent subsets. One subset (20% of the total training set compounds) was used for validation purposes, while the remaining four subsets (80% of the total training set compounds) were used to develop QSAR models. This procedure was repeated five times until each training compound was used for prediction once in a test set. The statistical parameters were computed after conducting the cross-validations for individual models.

Each model in this study generated the likelihood of an active assay response for a target compound.^40^ The model outputs used a sigmoid activation function to represent the predicted results as probability values between 0 and 1. The probability values were used to calculate the carcinogenicity probability, which estimates the toxicity probability of the compound. The consensus QSAR models were developed by averaging the predictions of individual models. Carcinogens and noncarcinogens were classified based on probability scores using an arbitrary yet commonly accepted threshold value of 0.5, like in previous studies.^52−54^ The QSAR modeling workflow and modeling algorithms can be found in our recent publication,^40^ and the Python code for the QSAR modeling workflow can be accessed at https://github.com/zhu-research-group/auto_qsar/.

### Universal Statistical Parameters and Metrics for Assay Selections and Modeling

The PubChem assays extracted for the compounds tested in the IRIS probe dataset were evaluated to determine the correlations between the individual assays and the carcinogenicity endpoint. First, these assays were selected based on the number of activity results across all IRIS compounds, and assays with less than five active results were removed. Then, we implemented Fisher’s exact test of independence using the assay responses to carcinogenicity.^22^ The assays with *p*-values less than 0.05 were relevant to carcinogenicity and selected for QSAR modeling. Prior to modeling, an equal number of compounds were randomly selected to balance the two classifications within each assay dataset.^55,56^

Previous studies demonstrated robust model performance and consistency of sampling-based approaches, such as the undersampling method.^57−59^ Compared to oversampling, undersampling can avoid overfitting the active compounds in the modeling procedure. Therefore, undersampling was used to balance the active/inactive ratio in the datasets of all assays.

The five metrics listed below were calculated for each QSAR model as the main criteria for evaluation. Sensitivity is the metric that evaluates the model’s ability to predict active compounds (eq 1); specificity is the metric that evaluates the model’s ability to predict inactive compounds (eq 2); the correct classification rate (CCR) is the average of sensitivity and specificity, representing the overall predictivity of the model (eq 3); and the positive predictive value (PPV) refers to the fraction of active predictions that were correctly labeled (eq 4). These four statistical parameters are commonly used to evaluate the predictivity of QSAR models.^22,47,60−62^
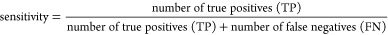
1
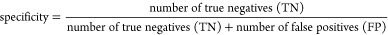
2
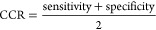
3
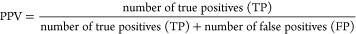
4

The carcinogenicity probability was calculated for each compound and respective assay endpoint (eq 5). This parameter estimates the ability to prioritize compounds with potential human carcinogenic activity, where *n* is the number of assays with consensus model predictions and *P*(A*i*) is the probability predicting the likelihood of a compound to be active in assay *i*.
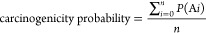
5

## Results and Discussion

### Modeling Workflow

**Figure 1:**
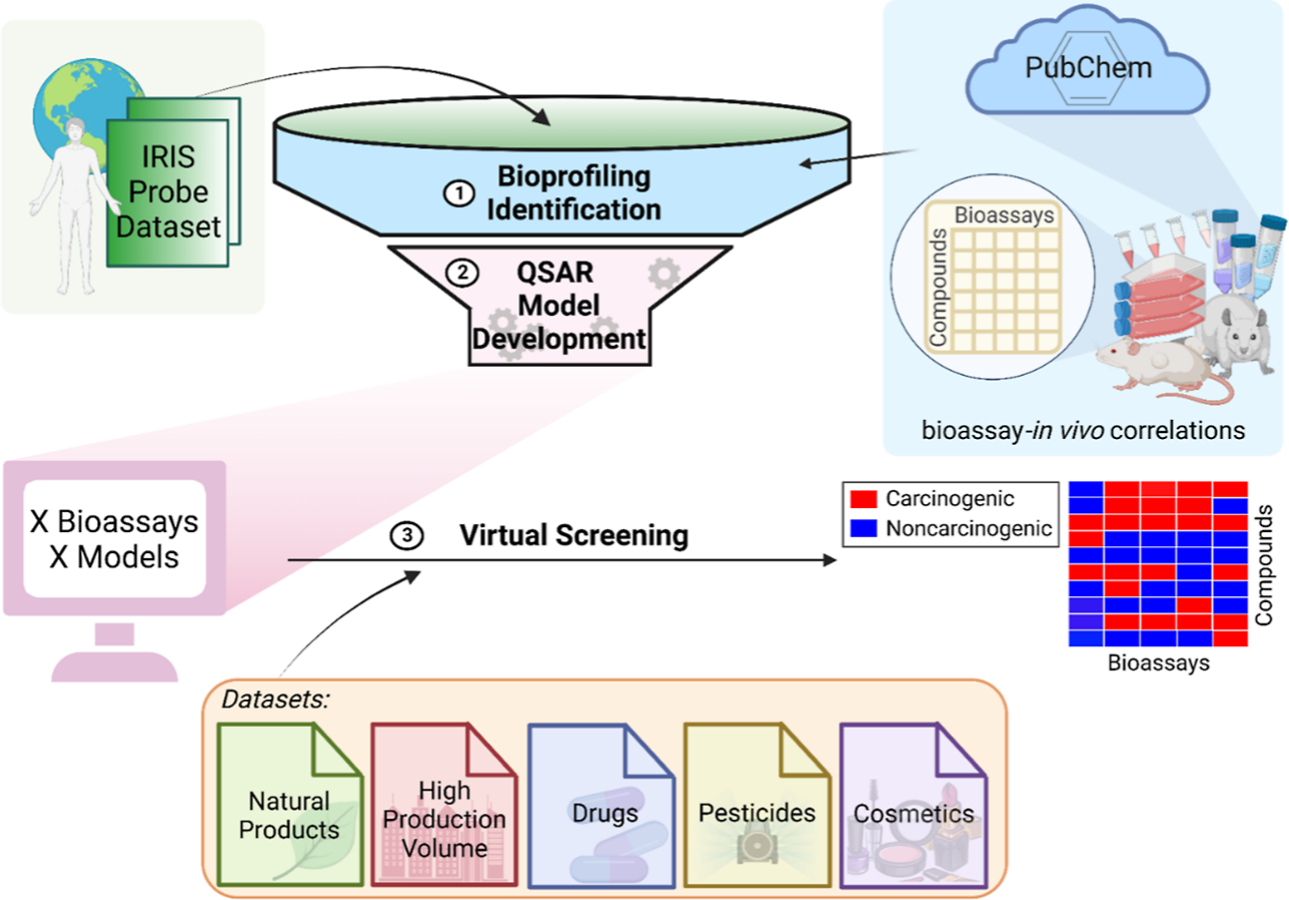
Schematic QSAR modeling workflow used in this study to model the carcinogenic potential of chemicals. The workflow consists of three steps: (1) training set generations by automatic bioprofiling, (2) QSAR model developments, and (3) virtual screening. Created with BioRender.com.

The modeling workflow, which lays the foundation of this study, is represented in Figure 1. First, the curated IRIS dataset was used as a probe to extract all PubChem assays containing response information (i.e., active, inactive, or inconclusive) for the probe compounds. Then, the extracted PubChem assays from the bioprofile were ranked by their correlations to human carcinogenicity. Eight assays were selected for QSAR model development based on the data available for the probe set compounds and the correlation between the assay responses and carcinogenicity. Five ML approaches were implemented in combination with 3 types of chemical fingerprints to develop 15 QSAR models for each assay. The resulting models were evaluated using 5-fold cross-validation. The consensus models yielding satisfactory cross-validation results were used to predict the five external datasets (i.e., drugs, pesticides, cosmetics, natural products, and high-production volume compounds) to prioritize potential carcinogens. Compared to classic QSAR modeling studies, an advantage of this workflow is the significantly enlarged training data by automatically data mining rather than using the original small training set. The whole procedure, including the generation of the enlarged training set of carcinogenic compounds (step 1), development of QSAR models (step 2), and prediction of new compounds (step 3), can be accomplished automatically with low computational cost.

### Generating Training Sets

The EPA’s IRIS dataset consists of identified human carcinogens, classified by human health assessments based on existing toxicity studies, demonstrating a causal relationship between chemical exposure and cancer. Every compound was classified based on the toxicity data from available animal studies while considering pivotal factors such as the mechanistic relevance, the number of studies, and the appropriate experimental protocols.^63−66^ Therefore, this dataset integrated various toxicity studies evaluated by experts to classify the human carcinogenicity of 485 compounds from a lifetime of oral exposure. After data curation, the probe dataset contained 342 unique compounds. Among them, 59 compounds were classified as human carcinogens, and the remaining 283 compounds were noncarcinogenic to humans following oral exposure (Table S1). While compounds in the IRIS dataset were not classified as human carcinogens with chronic oral exposure, some of these compounds may cause other toxicities. For example, resmethrin (CAS 10453-86-8) is noncarcinogenic to humans but was reported to have reproductive toxicity effects.^67^ Propargyl alcohol (CAS 107-19-7) is also noncarcinogenic to humans. However, this compound exhibits pathological renal manifestations and hepatotoxicity.^68^ In this study, the target endpoint was human carcinogenicity by oral exposure, and other toxicities induced by the same compounds were not considered.

**Figure 2:**
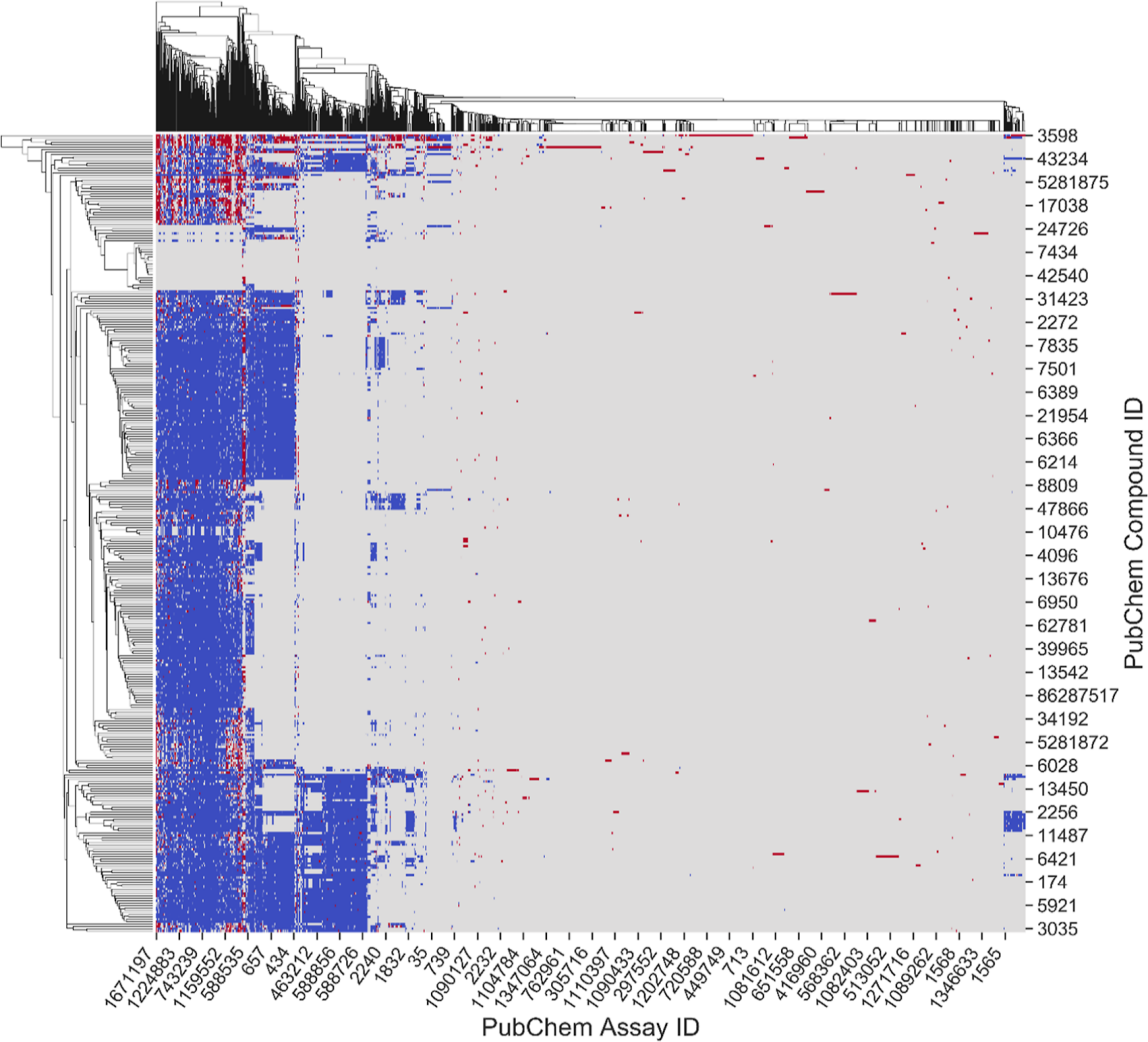
Bioprofile of 342 IRIS compounds consisting of 1971 PubChem bioassays. The heat map with hierarchical clustering aggregates testing results from each bioassay as “active” (red), “inactive” (blue), and “inconclusive/untested” (gray).

All 342 compounds were searched against the PubChem database to explore all available assays with test results for any of these compounds. Figure 2 represents the diverse landscape of the extracted data with abundant toxicity information for the probe compounds. The initial bioprofile for the probe compounds consisted of 1971 PubChem assays, and the extracted biological data for compounds in each assay were classified as active, inactive, and inconclusive (Figure 2). This initial bioprofile extracted by the 342 probe compounds contained 8128 active and 85,029 inactive results, greatly extending the data available for probe compounds. Not all assays are relevant to carcinogenicity, and the data gap (580,925 inconclusive/untested results) makes the initial profile unsuitable for modeling. Furthermore, the bioassay data exhibited biased results with significantly more inactive than active test results.^19,69,69−71^ Although this initial bioprofile contained large amounts of new data, an abundance of irrelevant data existed, and further optimizations were required before QSAR model development.

Fisher’s exact test was used to identify the assays with a significant association between activity and carcinogenicity. As a result, the assay responses of 25 assays have statistically relevant relationships to carcinogenicity (*p* < 0.05) (Table S2). Therefore, the identified 25 assays have the potential to be used to model carcinogenicity, and the QSAR models were developed using the data from these assays.

Each dataset of the 25 assays extracted from PubChem ranges from 738 to 7099 unique compounds (Table S2), including varying numbers of compounds from the IRIS probe dataset. The data curation process described above was used to remove duplicates and inorganic compounds that cannot be modeled within these datasets. Although the relationships between these 25 assays and the probe dataset are statistically significant (*p* < 0.05), assays with low predictivity for the actual carcinogens in the probe dataset are unsuitable for modeling purposes. To this end, each assay’s correlation (CCR) to carcinogenicity was calculated, where the carcinogenicity of existing probe compounds was compared to their assay responses. Golbraikh et al.^72,73^ indicated that the CCR of reliable QSAR models should be at least 0.65. Previous studies have shown that the same or similar CCR value thresholds are acceptable for reliable QSAR models.^74−76^ Therefore, a CCR value of 0.65 was used as the cutoff value to select the QSAR models in this study.

Eight assays with a CCR above 0.65 were retained to predict the carcinogenic potential of the compounds of interest, and the remaining 17 assays were removed. Many of these removed assays were protein target-specific and used an in vitro dose response for determining the endpoint activity. Efforts to bridge the gap between *in vitro* and *in vivo* activities require more advanced algorithms. For example, critical relationships among various assays were inferred using deep learning techniques to predict *in vivo* toxicity in a previous modeling study.^77^ These eight assays, along with their CCR, PPV, and coverage values for predicting the training set compounds, are represented in Figure S1. Coverage reflects the proportions of probe compounds tested in these assays, and the QSAR models can predict the remaining untested probe compounds. Although each assay correlation had low PPV values (i.e., between 0.33 and 0.63), the predictivity of carcinogens can be improved by combining multiple assay outcomes. All eight assay datasets contained more compounds than the IRIS probe dataset, ranging from 738 to 6985 compounds (Table S2). Therefore, instead of using probe compounds, which only contained 59 toxic compounds, the QSAR modeling based on these enlarged training data can be performed to generate better models.

Among the eight datasets, two were significantly biased, containing more inactive than active responses and vice versa (i.e., AID 1259408 and AID 1259411). Some of the selected assays were based on toxicity studies in animals. For example, carcinogenicity studies conducted in mice (AID 1199) and rats (AID 1208) measured the potency and detected tumor sites by toxicant exposure. Other selected assays, such as mutagenicity testing (e.g., AID 1194, AID 1259407, and AID 1259408), involved in vitro methods initially designed to predict carcinogenic activity.^78−81^ The other assays (i.e., AID 1189 and AID 1205) use male and female rodent cells to estimate the carcinogenic potential of a compound from tumor sites.^82,83^ While this study has presented a data-driven modeling workflow that can be used to expand the training data, it is still necessary to obtain additional *in vivo* toxicity data to conduct QSAR modeling and validate the predictions of human carcinogenicity.

Increasing the number of training set compounds makes the QSAR models applicable to a broader chemical space. The original eight assay datasets comprised a scope of compounds ranging from 738 to 6985 (Table S2). After the balancing procedure, these eight assay datasets ranged from 610 to 6985 unique compounds and were used to develop QSAR models. The chemical space of the probe set compounds and the selected training set assay compounds were generated based on the top 3 principal components from 206 two-dimensional descriptors using Molecular Operating Environment v.2019.01 (Chemical Computing Group Inc., Montreal, Canada). The overlap of the probe and training set chemical space demonstrates that the probe set and the training sets cover similar and broader chemical space (Figure S3). The combined probe and training sets suggest that the compounds used in the QSAR models represent the physicochemical properties used in the models. Furthermore, it can improve the interpretability of relevant toxicity mechanisms, emphasized in the Organization of Economic Co-operation and Development (OECD) principles for developing and validating QSAR models.^84^

### Quantitative Structure–Activity Relationship Modeling

Combinatorial QSAR models were developed for each PubChem assay by combining one of the three chemical fingerprints (i.e., ECFP, FCFP, and MACCS) and one of the five ML approaches (i.e., ADA, BNB, KNN, RF, and SVM). The combinatorial modeling resulted in 15 QSAR models for each assay. The OECD guidelines for risk-assessment QSAR validation published in 2007 state that models’ goodness-of-fit, robustness, and predictivity must be evaluated.^50^ An accepted method suggested by these guidelines is cross-validation,^50^ which has been shown to provide a reliable evaluation of a QSAR model’s performance.^51^ Therefore, all models were evaluated using a 5-fold cross-validation procedure.^51^ The resulting QSAR models showed acceptable predictivity using the 5-fold cross-validation procedure. The CCR values from the individual models ranged from 0.57 to 0.77.

**Figure 3:**
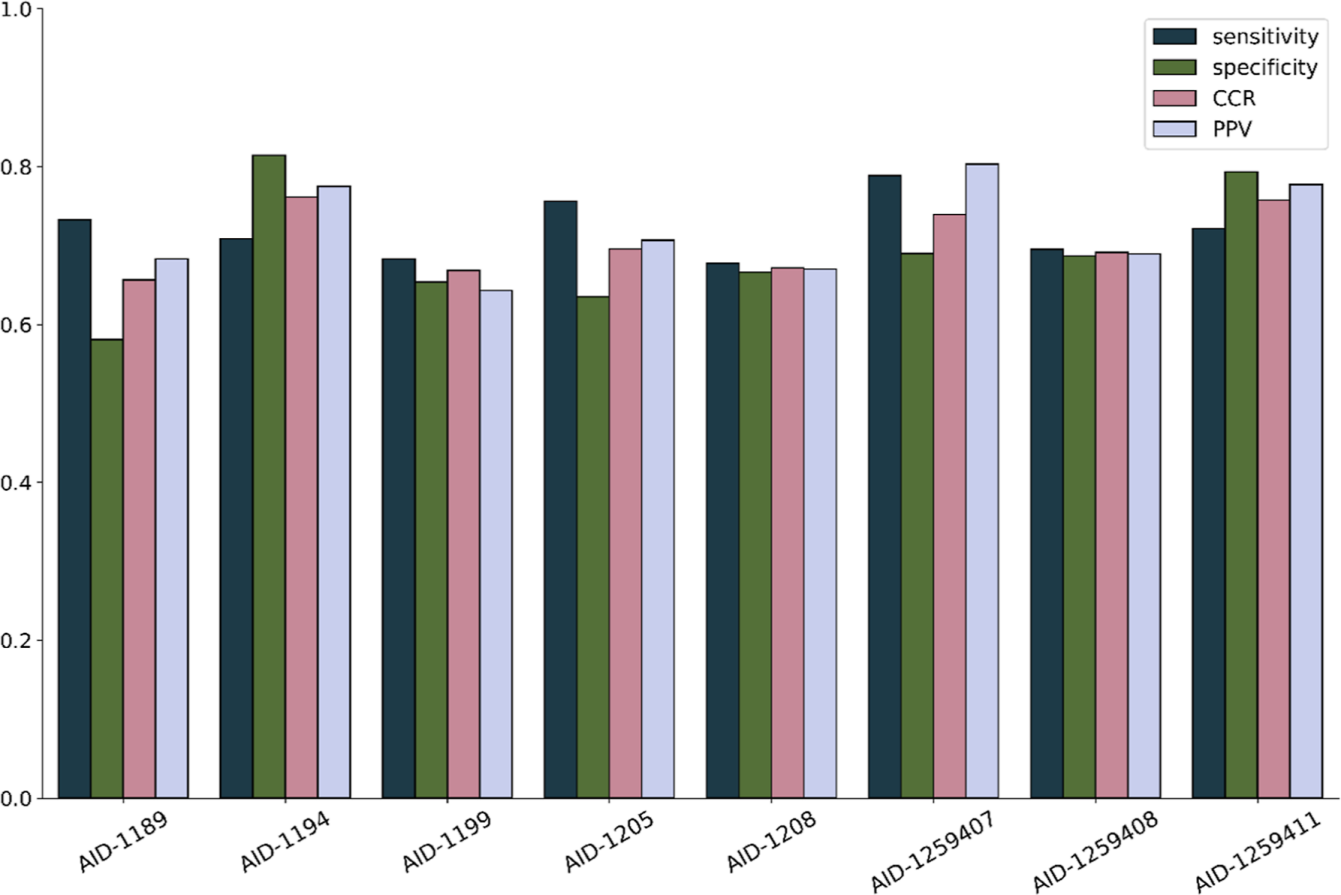
Performance of the consensus QSAR models developed for eight PubChem bioassays. Statistical evaluation of the consensus QSAR model was constructed by averaging 5-fold cross-validation prediction values from individual models, including the sensitivity (eq 1), specificity (eq 2), CCR (eq 3), and PPV (eq 4).

Consensus modeling, a weight of the evidence approach, has demonstrated its applicability in achieving similar to the best individual predictivity while leveraging predictions across feature spaces and algorithms.^20,21,24,62,85−88^ The consensus QSAR models were developed by averaging the predictions of individual models as described previously. As a result, the CCR values of the consensus models had higher performance, with CCR values ranging between 0.66 and 0.76 and PPV ranging between 0.64 and 0.80 (Figure 3).

### Virtual Screening

**Figure 4:**
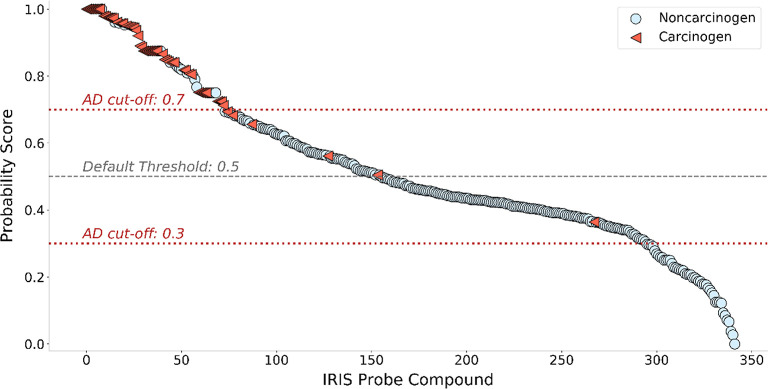
Ranking probe compounds in the IRIS dataset using carcinogenicity probability (eq 5) results. Red triangles represent human carcinogens associated with oral exposures (*N* = 59), and blue circles represent noncarcinogens (*N* = 283). The red dotted lines represent the applicability domain (AD) cut-offs. The gray dashed line represents the default threshold value of 0.5 to classify carcinogens/noncarcinogens based on the prediction values.

The consensus QSAR models were used to impute the missing data of the probe compounds against the eight assays. Then, the carcinogenicity probability was calculated for each probe training dataset compound using the combinations of experimental and predictive eight assay outcomes (eq 5). The experimental outcomes of assays are binary classifications with values of 0 and 1. However, if no experimental data available for probe compounds exists, the prediction outcome of an assay generated from consensus QSAR models is a value between 0 and 1. Finally, the probe compounds were ranked using the average carcinogenicity probability values (Figure 4).

In the context of regulatory compliance, a false negative compound indicates that the models failed to identify a carcinogen. In order to minimize the risk of false negatives, regulatory bodies often use multiple tests and evaluations to assess compliance. Similarly, our models incorporated an applicability domain component based on prediction confidence, determined by the consensus probability. This study’s applicability domain refers to the probability range in which the model is expected to provide accurate and reliable potential carcinogenicity results. For example, aniline (CAS 62-53-3) was predicted as a noncarcinogen with a probability of 37.3%. This approach is similar to using prediction confidence as an applicability domain in our previous modeling studies.^89−91^ By leveraging different QSAR models, each prediction is assigned a consensus probability value. The carcinogenic probability over 0.7 and the noncarcinogenic probability below 0.3 are used as the criteria to determine whether a prediction falls within the applicability domain. If the probability value of a compound was between 0.3 and 0.7 (i.e., aniline), the results of the model were considered unreliable or inaccurate. Figure 4 shows that establishing an applicability domain of the models can reduce or eliminate the false negative compounds. The change in the CCR between the model using a default classification threshold of 0.5 (CCR = 0.7) and the model using an applicability domain (CCR = 0.83) is an increase of 18.6%, suggesting that the use of an applicability domain improved the model’s performance.

Some false positives are potential carcinogens by other exposure routes, including hazardous chemicals for those disproportionally exposed in a workplace. For example, formaldehyde (CAS 50-00-0), an occupational toxicant, is a false positive prediction based on oral exposure but is a human carcinogen via inhalation.^92−95^ The other false positives, chloroform (CAS 67-66-3) and naphthalene (CAS 91-20-3), are also listed as potential carcinogens to humans when considering risk assessment by the inhalation route.^96−99^ Since the endpoint for modeling in this study is carcinogenicity through oral exposure routes (i.e., defined by the OSF in the IRIS dataset), this limitation can be corrected by extending the current endpoint.

Generally, QSAR models with small training sets have known limitations, such as small chemical space, activity cliffs, and susceptibility to overfitting. Even the most widely accepted computational models used in today’s industry settings have been developed with small datasets.^17^ This study aimed to expand the chemical space covered by carcinogenicity models and incorporate more support for predictions in the form of publicly available biological assay data. A primary limitation of this modeling approach is the selection of the relevant assays and thresholds for defining a toxic and nontoxic compound, in which such criteria are often arbitrary. However, the criteria can be adjusted based on the appropriateness and use case. It is acknowledged that the selected assays use animal studies to predict human carcinogenic potential, which has inherent limitations. Hence, this study intended to serve as a guide in this complex endpoint, recognizing the importance of prioritization and additional testing.

**Figure 5:**
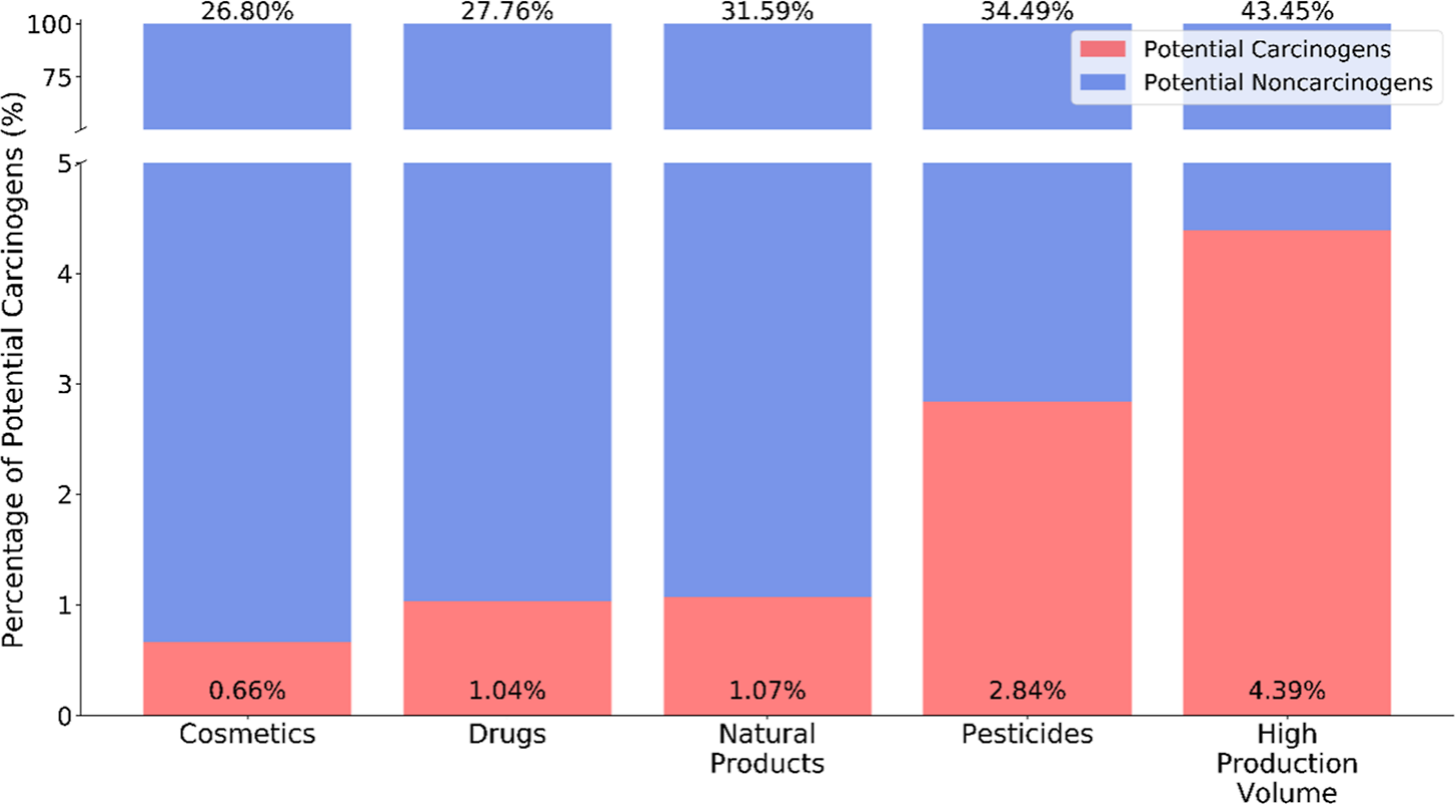
Distribution of compounds for five external screening datasets by the proportions of active predictions to total results from QSAR models (top) and the proportions of potential carcinogens to the total number of compounds (bottom).

The QSAR models were further validated by predicting five external datasets containing thousands of new compounds. First, overlapping compounds with the probe dataset were removed for each dataset to ensure that all the external compounds were new to the developed QSAR models. A profile of eight PubChem assays was created by obtaining the experimental testing results for all new compounds and using the consensus model predictions to fill the data gap by calculating the carcinogenicity probability (eq 5). The active predictions were obtained from the consensus QSAR model of the selected eight assays, and the predicted active was defined when more than half of all predictions were active. Based on the hypothesis that active predictions from these QSAR models indicate potential human carcinogenicity, an external dataset with more active predictions from the QSAR model will likely contain more potential carcinogens. As shown in Figure 5, cosmetics, drugs, natural products, pesticides, and high-production volume compounds have 26.8, 27.8, 31.6, 34.5, and 43.5% active/carcinogenic predicted values among all predictions, respectively. Therefore, the prediction results were consistent with the nature of these chemical classes (Figure 5). Cosmetics and drugs, which are explicitly tailored to humans and are strictly regulated, were predicted to have only a small proportion of potential carcinogens (i.e., 0.7 and 1.0% of total compounds, respectively). Natural products, considered relatively safe and widely used in Chinese herbal medicine,^100^ are also predicted to have a low proportion (1.1%) of potential carcinogens. On the contrary, pesticides and high-production volume compounds, which were predicted to contain relatively high ratios of potential carcinogens (i.e., 2.8 and 4.8%, respectively), have been reported to be relevant to cancer incidence and mortality.^101,102^

As described above, 227 compounds were identified as potential carcinogens within these 5 external sets. Among these compounds, 32 (14%) showed active responses across all 8 assays, and they are ranked with the highest probability of being carcinogens. The top-ranked potential carcinogens in these five datasets are shown in Table 1. These compounds were found to be registered as carcinogens in the European Chemicals Agency (ECHA) classification and labeling (C&L) inventory. The C&L inventory by the European Union contains chemical information, including known or presumed carcinogenic potential for humans, for regulatory agencies.^103−105^ The chemicals’ hazard statement codes (i.e., H350 and H351) obtained from this resource indicate general concern for chemical carcinogenicity (Table 1). Annex I of the ECHA legislation is a list of compounds that are subject to the strictest controls of the European Union’s chemical regulation.

**Table 1:**
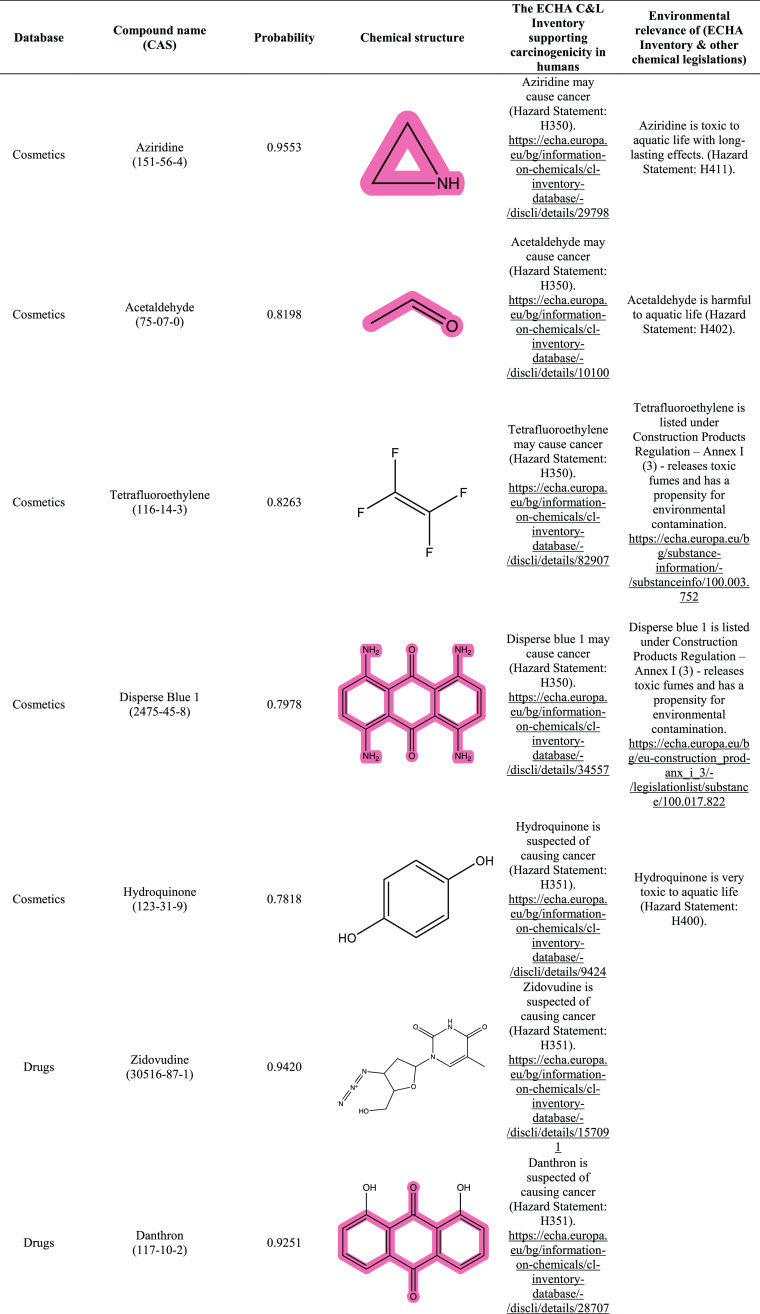

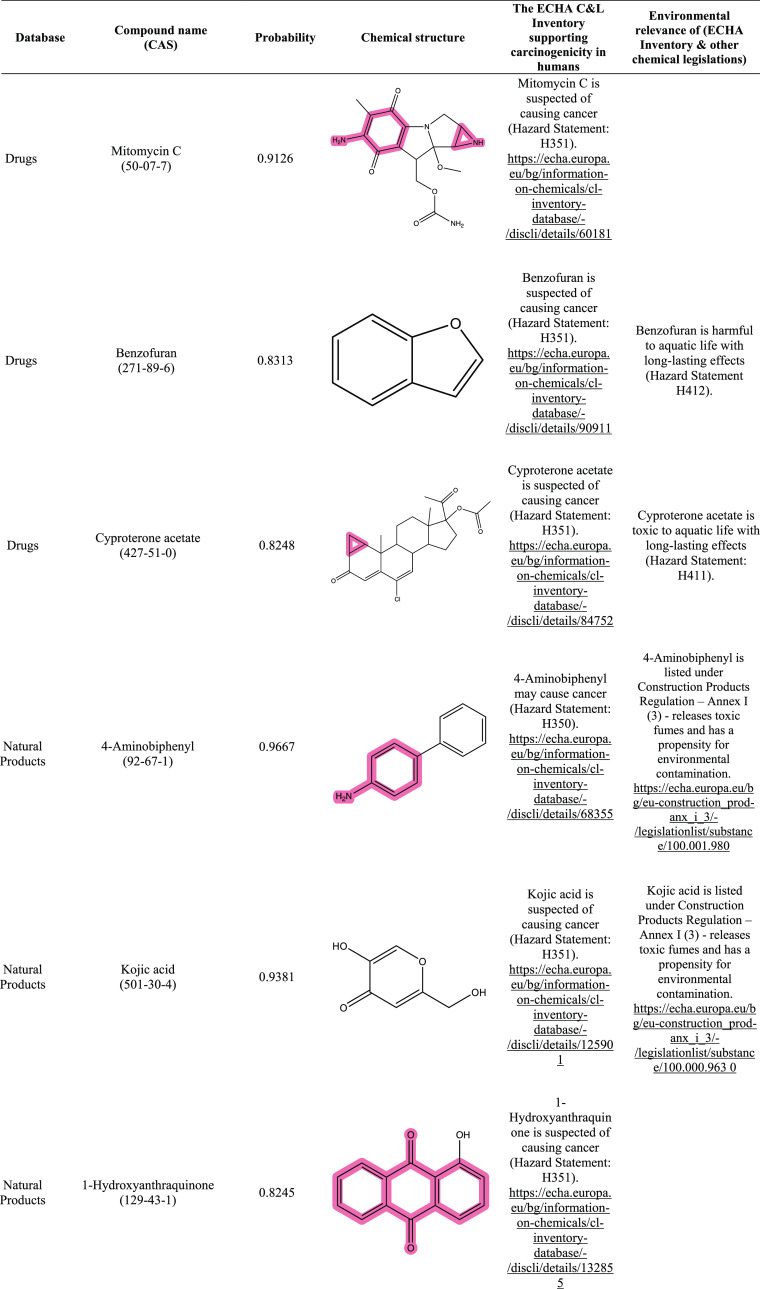

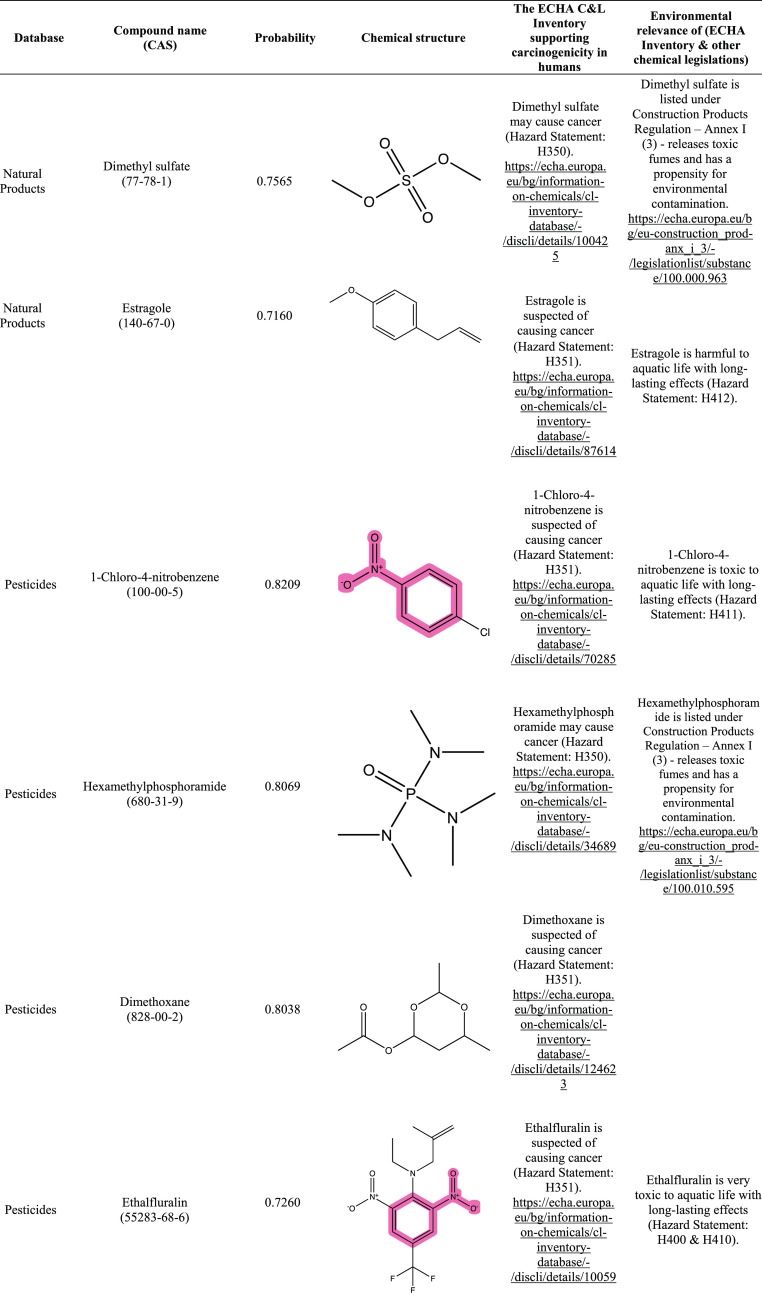

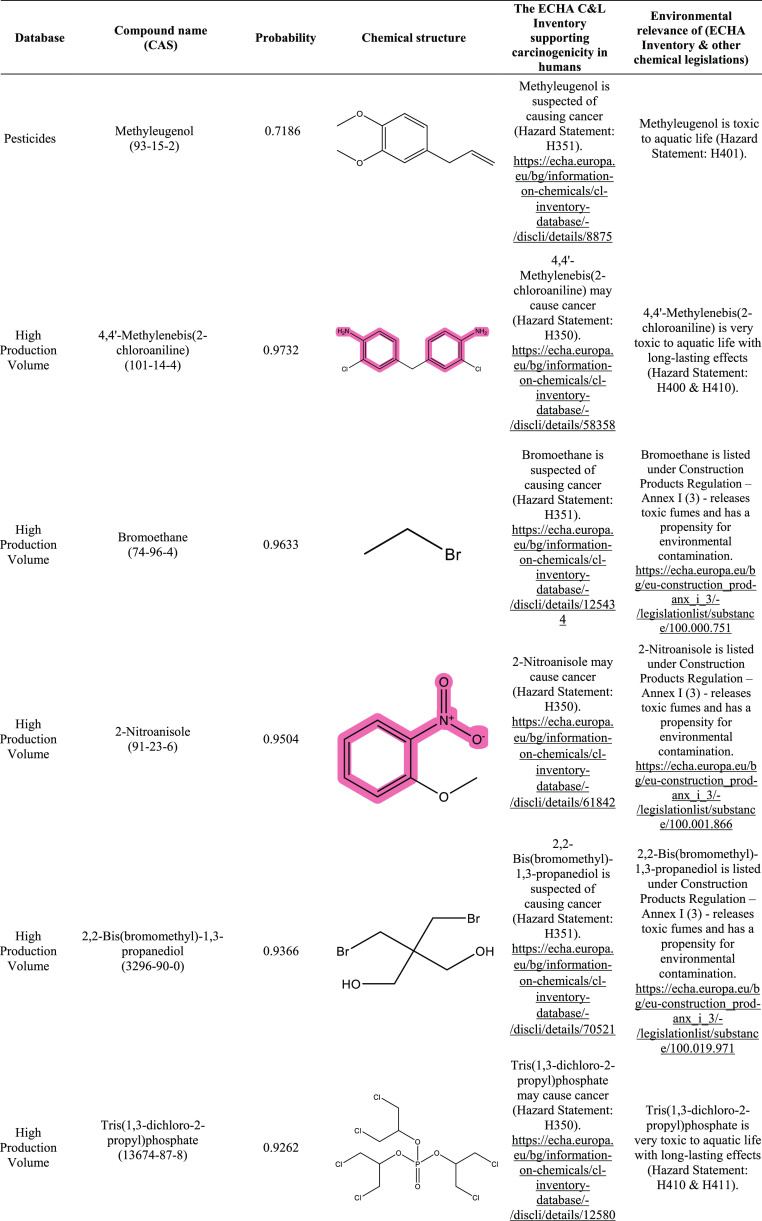
Top-Ranked Potential Carcinogens in Five External Datasets Based on Probability Scores

Providing a deeper understanding of the potential chemical risks from different environmental exposures (e.g., manufacturing, use, and disposal) can improve the safeguarding of human health. Table 1 shows the compounds reported by the ECHA and their associated hazard statements indicating acute or chronic toxic effects on aquatic life at relatively low concentrations. A potential carcinogen, such as danthron, may have physiochemical properties that can contribute to bioaccumulation and biomagnification even after being banned and disposed of.^106−109^ Studies have suggested that danthron and other compounds may accumulate in the tissues of animals,^110−116^ which raises concerns and could have potential implications for human health if entering the food chain. Consequently, even if a chemical has been flagged as a carcinogen and prohibited from further use, the distribution of ecological systems also needs to be studied. As shown in Table 1, the top-ranked potential carcinogens from five external datasets all have carcinogenicity concerns from the ECHA, which validated the applicability of our QSAR models.

Interestingly, evaluating the potential carcinogens revealed the same chemical scaffolds that may act as toxicophores. Some potential toxicophores were highlighted based on previous carcinogenicity studies. For example, formerly used in fabric dye—disperse blue 1 (CAS 2475-45-8), a drug no longer used as a stimulant laxative—danthron (CAS 117-10-2), and an obsolete intermediate in the production of dyes and pharmaceuticals—1-hydroxyanthraquinone (CAS 129-43-1) are anthraquinone derivatives. Anthraquinones have been reported to induce carcinogenic responses in animals and humans.^110−112^ These identified potential carcinogenic compounds share similar structural components with polycyclic aromatic hydrocarbon, wherein ketone groups are found in the central ring and contain a phenol group. Other top-ranked potential carcinogens, aziridine (CAS 151-56-4), mitomycin (CAS 50-07-7), and cyproterone acetate (CAS 427-51-0), have three-membered rings within their structures. More specifically, aziridine and mitomycin contain three-membered heterocyclic amines formed from charred meats and have been reported to pose human carcinogen-induced DNA damage.^117,118^ A literature search revealed the presence of several shared toxicophores (e.g., aromatic amines, aromatic nitro compounds, and acetaldehyde) among the highest-ranked compounds for potential carcinogenicity.^119−123^

Taken together, Table 1 shows the results of the QSAR modeling approach and demonstrates the carcinogenic potential of chemicals that increase the chance of developing cancer in humans, supported by the ECHA. Using the QSAR modeling approach, users can evaluate similar chemical structures, which provides more information about the compounds and concurrently enhances their understanding of the chemical relevance and applicability domain. This precedent supports the fundamental principle in QSAR that chemicals with similar structures exhibit similar properties, including biological activities.^124−126^

With the goal of assessing the predictivity reliability of the developed models in this study, a benchmark study was conducted to compare the performance of our models with that of VEGA-CAESAR (https://www.vegahub.eu/portfolio-item/vega-qsar/, assessed October 20, 2022) using the IRIS probe dataset (Table S3). The analysis revealed that our approach could ensure reliable statistics to predict carcinogenic compounds (PPV = 1.0) compared to the existing model (PPV = 0.26). Our modeling approach allows an opportunity to complete highly tailored mechanistic studies based on specific endpoints and uncovers a larger chemical space using a three-step guideline.

Using the automatic data-driven QSAR modeling approach developed in this study, an initial dataset with a limited number of compounds can significantly expand to multiple relevant bioassay datasets with many more compounds. This approach can be applied to insufficient training sets for modeling complex toxicity endpoints. Additionally, we incorporated an applicability domain for the predictive classification model to improve our model predictions’ accuracy in accordance with the OECD guidelines.^50^ With this consideration, the results show that QSAR models with defined applicability domains improve prediction confidence. This approach resulted in the development of models with eight assays (i.e., only eight toxicity endpoints related to carcinogenicity). Despite using a limited number of assays, the resulting QSAR models showed good predictivity for the original toxicity endpoint of human carcinogenicity from chronic oral exposure caused by various compounds. As this modeling study aimed to prioritize potential human carcinogens from long-term oral exposure, the criteria for selecting the assay data and defining the prediction-based carcinogens warranted the resulting models to predict the toxic compounds. Depending on the target toxicity endpoint, these arbitrary criteria can be modified and defined for future studies.

This study described a data-driven computational approach that applies public data to expand a small dataset with only 59 orally exposed human carcinogens and 283 noncarcinogens into 8 large training sets of up to 6985 compounds, which is better suited for QSAR modeling. Instead of modeling a single small dataset for a single endpoint, a total of 120 QSAR models were developed for 8 datasets with 8 relevant endpoints. These models were applicable to predict the target endpoint of human carcinogenicity by chronic oral exposure. The consensus models, which averaged the results of the individual QSAR models, showed good predictivity and were used to identify potential human carcinogens from chronic oral exposure. By virtually screening 5 external datasets of different chemical classes, 227 potential human carcinogens were identified. The top-ranked compounds were also reported as known or suspected carcinogens in the European Union market. Modeling complex toxicity endpoints using a small dataset is normally not feasible. Our approach can answer this challenge by creating a larger training set by mining public data, which can be applied to computational toxicology studies in the current big data era.

## Supporting Information Available

Correlations between bioassay responses and human carcinogenicity by the oral route of exposure, distributions of active (bottom) and inactive (top) results in assay datasets, chemical space of the IRIS dataset (*n* = 342), and training set compounds in assay datasets (*n* = 14,728) (PDF)
EPA’s Integrated Risk Information System (IRIS) dataset, identified carcinogenicity-related PubChem assays, and evaluation of benchmark QSAR models to predict carcinogenicity (XLSX)
