# Supplementary material for: Data-Driven Quantitative Structure–Activity Relationship Modeling for Human Carcinogenicity by Chronic Oral Exposure
Source: Environ Sci Technol. 2023 Apr 11;57(16):6573–88. doi: 10.1021/acs.est.3c00648 (PMC10134506; doi:10.1021/acs.est.3c00648)
Supplement: Supplementary file 1 — es3c00648_si_001.pdf [file es3c00648_si_001.pdf]

Supporting Information

Data-Driven Quantitative Structure-Activity Relationship (QSAR) Modeling for Human Carcinogenicity by Chronic Oral Exposure

Elena Chung[1], Daniel P. Russo[1], Heather L. Ciallella[2], Yu-Tang Wang[3], Min Wu[4], Lauren M. Aleksunes[5], Hao Zhu[1]*

[1]Department of Chemistry and Biochemistry, Rowan University, 201 Mullica Hill Road, Glassboro, New Jersey 08028, USA.

[2]Department of Toxicology, Cuyahoga County Medical Examiner's Office, 11001 Cedar Avenue, Cleveland, Ohio 44106, USA.

[3]Institute of Agro-Products Processing Science and Technology, Chinese Academy of Agricultural Sciences/Key Laboratory of Agro-Products Processing, Ministry of Agriculture, Beijing 100193, China.

[4]School of Life Science and Technology, China Pharmaceutical University, No. 24, Tong Jia Xiang, Nanjing 210009, China.

[5]Department of Pharmacology and Toxicology, Rutgers University, Ernest Mario School of Pharmacy, 170 Frelinghuysen Road, Piscataway, New Jersey, 08854, USA.

Corresponding Author

*Hao Zhu, 201 Mullica Hill Road, Department of Chemistry and Biochemistry, Rowan University, Glassboro, New Jersey 08028; Telephone: (856) 256-4500; Email: zhuh@rowan.edu

Table of Contents

Supplementary Figure 1. Correlations between bioassay responses and human carcinogenicity by the oral route of exposure. Correlations are shown as the correct classification ratio (CCR, Eq. 3), positive predictive value (PPV, Eq. 4), and coverage.

Supplementary Figure 2. Distributions of active (bottom) and inactive (top) results in assay datasets.

Supplementary Figure 3. Chemical space of the IRIS dataset (n = 342) and training set compounds in assay datasets (n = 14,728). Chemical space plot on A) the probe IRIS dataset compounds (green dots represent active, and yellow dots represent inactive compounds) and B) on the training set compounds in assay datasets (red dots represent active, and blue dots represent compounds.

Additional File: Excel spreadsheet containing Supplementary Tables 1 – 3.

Supplementary Materials

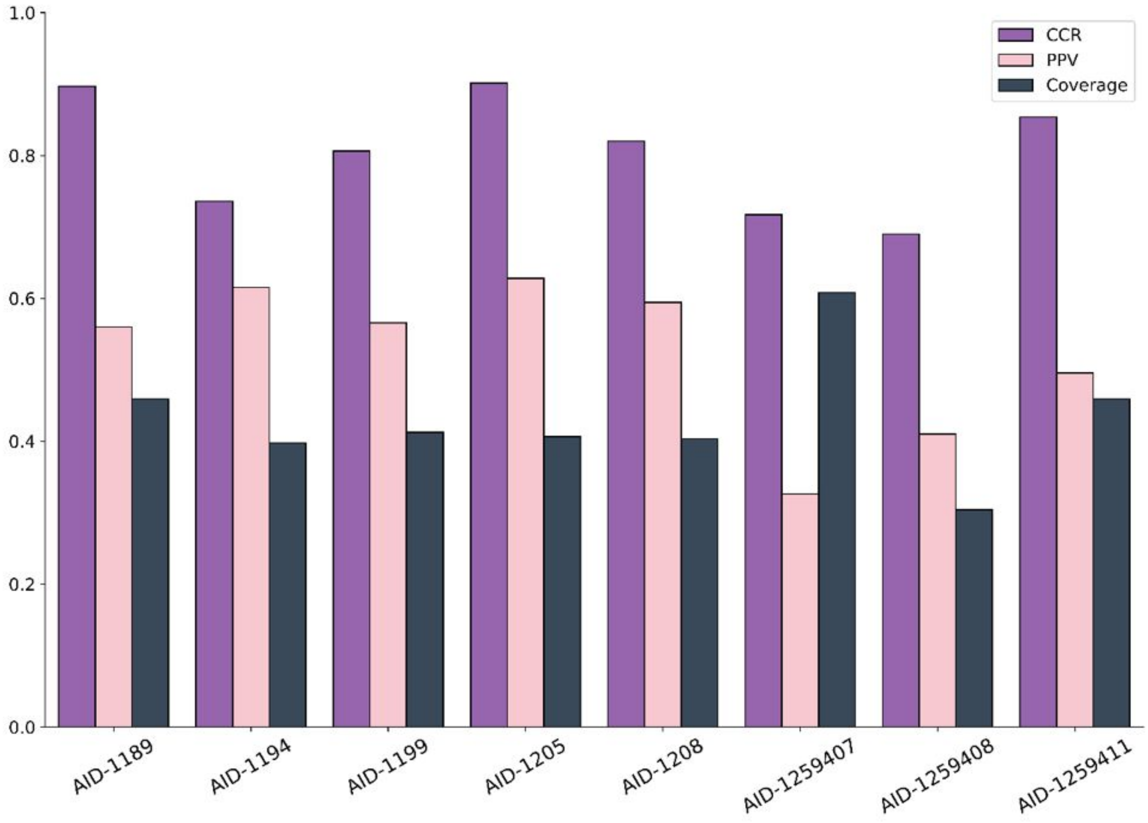

Supplementary Figure 1. Correlations between bioassay responses and human carcinogenicity by the oral route of exposure. Correlations are shown as the correct classification ratio (CCR, Eq. 4), positive predictive value (PPV, Eq. 5), and coverage.

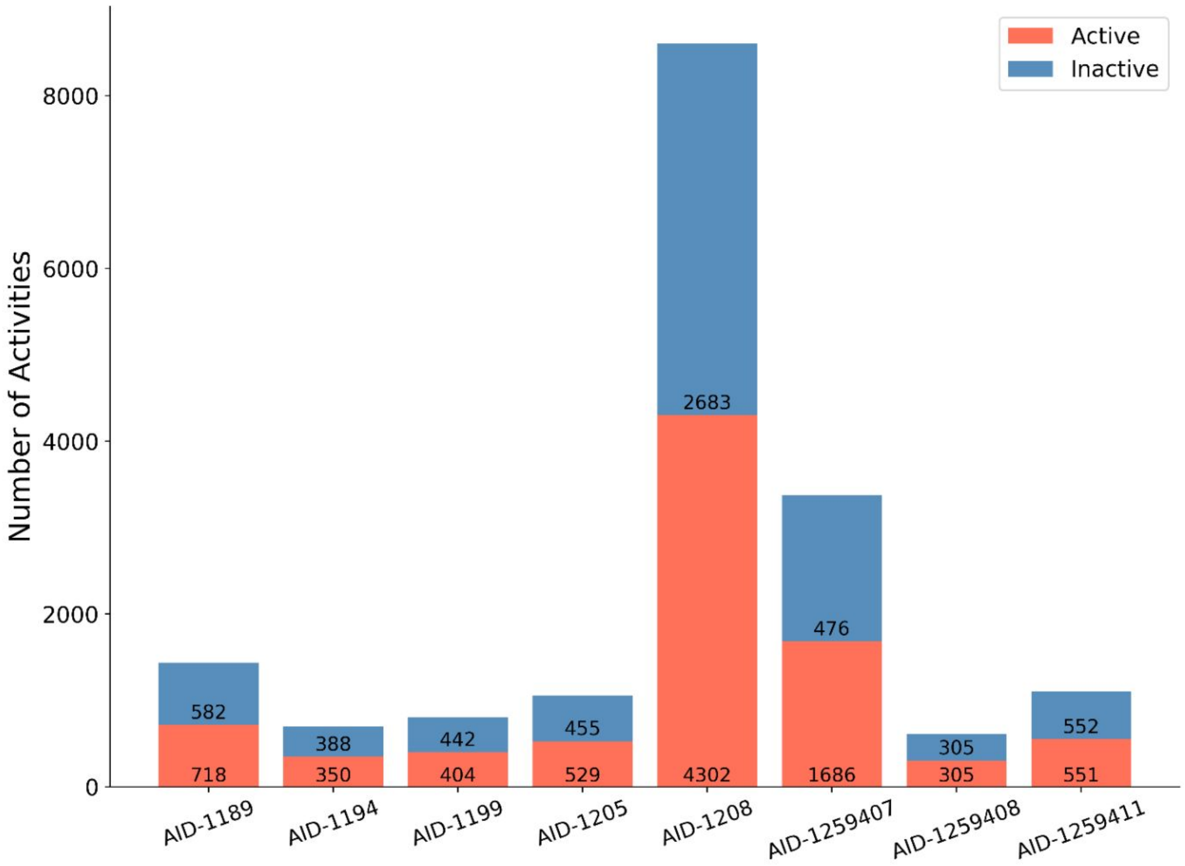

Supplementary Figure 2. Distributions of active (bottom) and inactive (top) results in assay datasets.

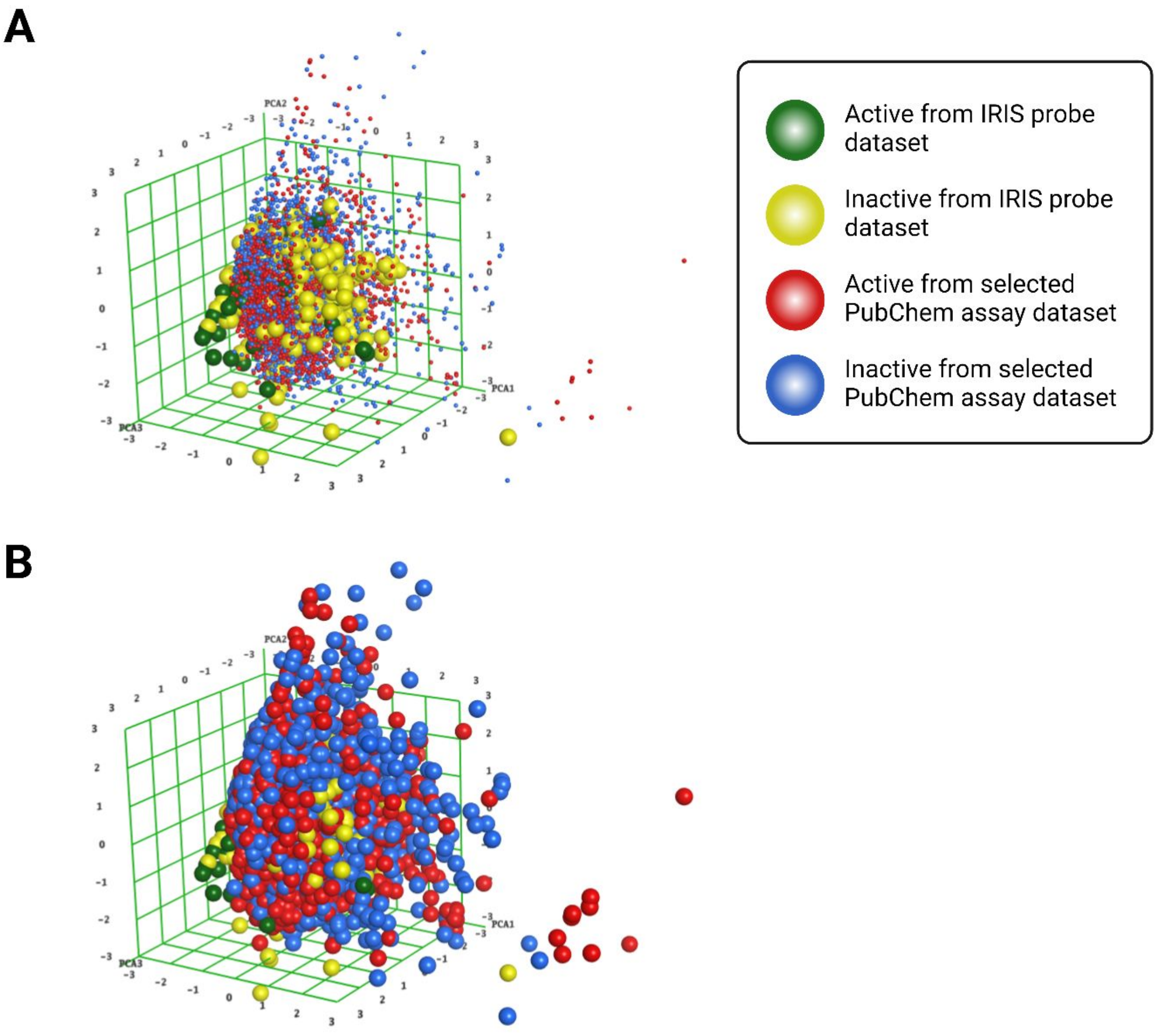

Supplementary Figure 3. Chemical space of the IRIS dataset (n = 342) and training set compounds in assay datasets (n = 14,728). Chemical space plot on A) the probe IRIS dataset compounds (green dots represent active, and yellow dots represent inactive compounds) and B) on the training set compounds in assay datasets (red dots represent active, and blue dots represent compounds.
